# The experience of work-life balance across family-life stages in Switzerland: a cross-sectional questionnaire-based study

**DOI:** 10.1186/s12889-015-2584-6

**Published:** 2015-12-24

**Authors:** Ariane G. Wepfer, Rebecca Brauchli, Gregor J. Jenny, Oliver Hämmig, Georg F. Bauer

**Affiliations:** Institute of Epidemiology, Biostatistics and Prevention, University of Zurich, Hirschengraben 84, CH-8001 Zürich, Switzerland

**Keywords:** Gender, Family-life stages, Part-time work, Gendered division of labor, Work-life balance

## Abstract

**Background:**

The division of paid and unpaid labor in families continues to be highly gendered with men doing more paid work and women doing more unpaid care work. This is especially true for life stages with young children. Our study investigates the subjective experience of demands in the work and the private domain and the experience of work-life balance across family-life stages as a consequence of this gendered division of labor.

**Methods:**

We used data from a survey study on work-life issues and health in four large companies in Switzerland (*N* = 3664).

**Results:**

In line with our hypotheses, subjective work and private demands were predicted by an interaction of family-life stages and gender. Specifically, during the primary child-rearing family-life stages, women experience more private demands than men while men experience more work demands, regardless of level of employment. Furthermore, women who work part time experience more work-life balance than women who work full time and more than men who work part or full time during the primary child-rearing family-life stages.

**Conclusions:**

Results are discussed in terms of a gendered work-life experience across the life course and the need for part-time work for both genders. Finally, conclusions are drawn concerning our results’ implications for public health considerations.

## Background

The link between work-life balance and health has been confirmed by much research, using diverse research approaches in many different samples and different national contexts [1–4]. Thus, it can be argued that the experience of balance between work and non-work life as an indicator of well-being and health is of public health interest [2] – not least because it is highly gendered (see below). Work time, gender and family-life stage have long been recognized and discussed as important determinants of the work-life experience [3, 4] but few studies have looked at their interactive effects [3, 5]. Research has found family-life stage to be more relevant than biological age when predicting people’s experience at the work-life interface [6]. The current paper aims to investigate the subjective experience of work-life balance as a result of the gendered distribution of paid and unpaid work across the life course in Switzerland. Due to comparably high female labor force participation and a high percentage of part-time workers, Swiss data is suitable to investigate the dynamics of gender and work hours in shaping the work-life experience across family-life stages.

### Today’s work-life experience

The workforce has changed considerably in western societies in the past 40 years [7]. Social changes have given rise to a much broader participation of women in the labor market [8]. In Switzerland, the employment rate of women aged 15 to 64 has risen from 66.4 % in 1991 to 74.1 % in 2013 [9]. The employment rate of men aged 15 to 65 has dropped from 90 % in 1991 to 83.9 % in 2013 [9]. The employment rate of mothers with children under 15 was 57.5 % in 1991 and 74.3 % in 2013. At the same time, the employment rate for fathers with children under 15 was 98.2 % in 1991 and 94.7 % in 2013.

While the workforce has changed, the expectations of the workplace have not [10]. Full-time work is still considered the norm [11] and often opportunities and perks hinge on full-time availability. The “ideal worker” norm comprises full-time availability, mobility, high qualifications, a strong work orientation and the readiness to put work first [12]. This norm stems from a time when most workers [13, 14] were men and could rely on a full-time homemaker (usually a wife) to manage the household and do the unpaid care work [10]. Today, most families do not have the backup of a full time homemaker. Statistics for Switzerland shows that in 2013 only 24.1 % of women with a partner and children under 15 were not part of the workforce. Among fathers with a partner and children under 15 this rate was 2.8 %, including retirees [9]. Thus the “ideal worker” norm is outdated and not in line with today’s workforce and its needs, yet it continues to shape the expectations of employers and thereby our working conditions [10].

Apart from large time commitment to work, there is evidence for an intensification of work through heightened work pressure and new technologies [13, 14]. Confronted with high demands and expectations in the work and the private domain, many families with young children experience extensive time pressures and a sense of imbalance or misfit [10, 11]. In a representative study on the Swiss working population, Hämmig, Gutzwiller, and Bauer [1] found that 12.5 % of the study population indicated experiencing high or very high work-life conflict. Living with dependent children was a significant predictor of work-life conflict for both sexes.

Across Europe and the USA, different countries have introduced a range of policies to foster the reconcilability of work and non-work life, especially for families. In the US, it is mainly up to employers to establish working conditions that support the reconcilability of work and non-work life for working families [15]. In Europe, and especially in the northern European countries, policies on a national level are more commonly found [16–18]. Still, national policies and welfare state regimes vary greatly across Europe and research shows marked differences in female labor-force participation and part-time work [16, 18–21]. The division of paid and unpaid labor between men and women with dependent children is shaped by a combination of national policies, organizational practices and societal and personal beliefs and expectations [21, 22]. In Switzerland, the combination of low state support for women’s labor force participation, together with expensive childcare and relatively high salaries leads to the neo-traditional model (men work full time, women part-time) being most commonly found [23]. In 2013, in 51.8 % of couple households with at least one child under 15, the male partner was full-time employed while the female partner was working part time [24]. Compared to other European countries, these part-time jobs often comprise very few hours. Figures from 2013 show that of the 61.8 % of mothers with children under 15 who work part time, more than half work less than 50 % [24]. As a result, the division of housework and childcare in families with dependent children follows a largely traditional pattern. While the overall amount of hours spent in paid and unpaid work differs by children’s age but is very comparable for men and women, figures from the SAKE 2013 show that women still do the bulk of the house and care work. Even though fathers nowadays are more involved in house work and child care, the great majority of families say that mothers are chiefly responsible for managing the household [9].

As a consequence, the work biographies of men and women differ markedly. For men, the norm still is continuous full-time employment from the entry into the workforce until retirement. The norm for women is part-time work or even a temporary exit from the workforce when children are born. In fact, only few women go back to full-time employment, even after their children are grown [25]. In an analysis of the situation of working families in the US, Moen and Sweet [11] discovered a similar situation and discussed this phenomenon under the term of the “gendered life-course”. Due to the reasons given above, the differences between men’s and women’s roles and experiences in the work and the private domain are very pronounced in Switzerland when compared to the northern European countries. In this study, we seek to investigate the subjective, experiential level of these demographic differences across the life course. We focus on the experience of subjective work and private demands and work-life balance.

### Family-life stages

The concept of *family-life stages* [26] has been used previously to explore how the experience at the work-life interface differs across the life course [6, 27] for men and women [3]. Family-life stages describe the family-life cycle divided into a sequence of consecutive stages, characterized by certain roles and demands on time and energy in the private domain as well as in the work domain [26, 27]. For a literature review of family-life stage-specific demands see Erickson et al. [27]. In our current study, we distinguish 5 family-life stages: (1) young adults without children, (2) parents of preschoolers, (3) parents of primary school-age children, (4) parents of children 12+ and (5) adults with grown children. Our operationalization follows Hill et al. [5] in using the age of the youngest child of a family to define family-life stages. As stated above, family-life stages are much more revealing than biological age when investigating work-life balance and its development over the life course. This fact is highly relevant when public health interventions are planned and implemented aiming at the elimination of inequalities concerning work-life balance and, in consequence, concerning well-being and health.

### Work and private demands across family-life stages

Research confirms that *non-work life demands* on time and energy are highest in family-life stages with young children at home and lowest in family-life stages without children and after children have left home [28, 29].

*Work demands* are theorized to partly parallel private demands [28, 30] and there is empirical support for this parallel trend across the life course [5, 27]. High private demands during the primary child-rearing years coincide with the heightened work demands of the primary career-building years [3, 28, 31]. Expressions such as “time squeeze” and “rush hour of life” have been used to describe this phenomenon [31, 32].

### Work-life balance

The concept of *work-life balance* captures the reconcilability between the work and the private domains and can be described as “an overall interrole assessment of compatibility between work and family roles” (p. 703) [33]. Unlike work-life conflict and enrichment, it is not about causal influence of one domain on the other [33]. There is far less research on work-life balance than on conflict and enrichment. It has sometimes been equated with low work-life conflict and high enrichment [34]. Lately though, researchers have begun to view work-life balance as a construct in its own right [33]. Greenhaus and Allen [35] discuss work-life balance as an outcome of satisfaction with and effectiveness in different life roles in accordance with one’s life values. Across the life course, the relative importance of work and family roles and therefore life values are likely to change. Therefore, work-life balance can be seen as an indicator of satisfaction with the extent to which people can live in accordance with their current life values. Furthermore, research has established its discriminant validity together with work-life conflict and enrichment [36].

## Study aims & hypotheses

Given the actual gendered division of paid und unpaid work in families in Switzerland, our current study seeks to investigate men’s and women’s subjective experience of demands in the work and private domain across the life course, as well as the experience of work-life balance. Special attention is given to family-life stages with young children. We do this in a big, diverse sample of employees from four large Swiss companies.

Previous studies on the reconcilability of work and non-work life in Switzerland have investigated different demographic predictors separately [1, 37]. In this study we look at the interactive effect of family-life stage, gender and level of employment, because these factors are not distributed at random but highly correlated. As discussed above, the employment pattern of men and women across family-life stages reflects a complex interplay of normative believes and expectations at the personal and the societal level, work practices and benefits at the organizational level, and policies and welfare state regimes at the political level. To understand the subjective experience at the work-life interface associated with this employment pattern across the life course, it is instructive to look at interactive effects of demographic variables.

Instead of focusing on conflicts or imbalance between work and non-work life, as previous Swiss studies have done [1, 37–39], we look at work-life balance as a more global assessment of reconcilability of work and non-work life.

In a first step, we investigate how family-life stage, gender and level of employment influence the experience of work and private demands. We expect *work demands* to be highest during family-life stages 2 and 3, because the career-building years tend to coincide with the primary child rearing family-life stages. Due to gender role expectations and related actual task distribution, we assume that, compared to women, men experience more work demands during the primary child rearing family-life stages, regardless of their level of employment.

### Hypothesis 1: work demands

- Work demands are highest during family-life stages 2 and 3.
- Men’s work demands are higher than women’s in family-life stages 2 and 3.

Moreover, we expect that *private demands* will be highest during the primary child-rearing years (family-life stages 2 and 3). Due to gender role expectations and related actual task distribution, we further assume that, irrespective of level of employment, women’s private demands will be higher than men’s during the primary child rearing family-life stages.

### Hypothesis 2: private demands

- Private demands are highest during family-life stages 2 and 3.
- Women’s private demands are higher than men’s in family-life stages 2 and 3.

In a second step, we investigate how family-life stage, gender and level of employment influence the experience of work-life balance. We expect *work-life balance* to be lowest during the primary child rearing family-life stages (family-life stages 2 and 3), because work and private demands are highest during those stages, which makes it difficult to achieve a sense of balance and reconcilability. Furthermore, we hypothesize that in family-life stages 2 and 3 women’s part-time work affords them a better work-life balance as compared to men, because on average women work fewer hours.

### Hypothesis 3: work-life balance

- Work-life balance is lowest during family-life stages 2 and 3.
- In family-life stages 2 and 3, women who work part time experience a better work-life balance than women who work full time or men who work part- or full time.

## Methods

### Data

The data was collected in a survey study on work-life issues and health at four large companies in Switzerland in various sectors: healthcare, banking, insurance, and logistics. We used information on age, number of children living at home and age of youngest child living at home to classify participants into five family-life stages. The original sample comprised 6091 participants. A subsample of *N* = 3664 could be classified into our five family-life stages. The other participants had missing values in relevant demographic variables or didn’t fit our specifications for family-life stages. There were less than 5 % missing values in all dependent study variables. Following Schafer & Graham’s [40] recommendations, missing values were replaced using maximum-likelihood estimation.

### Ethical aspects

The anonymity and confidentiality of participation in the study were ensured. Furthermore, participants voluntarily agreed to take part in the study and gave their consent prior to completing the questionnaire. All participants were allowed to complete the questionnaire during working time. Since the data collection was completely anonymous, according to the ethics commission of the Canton of Zurich, no approval of an ethics committee was necessary.

### Variables and measures

#### Work demands

A single item was used to obtain a global rating of subjective work demands. The item we used read: ‘How high is the intensity of your overall work demands?’ Participants rated the intensity of their overall work demands on a Likert-scale ranging from 1 = *very low* to 5 = *very high*.

#### Private demands

A single item, reading, ‘How high is the intensity of your overall private demands?’ was used to obtain a global measure of subjective private demands. Participants scored their overall private demands on a Likert-scale ranging from 1 = *very low* to 5 = *very high*.

#### Work-life balance

Two self-developed items were used to measure work-life balance. The items read ‘I feel the relation between my work and my non-work life is optimal’ and ‘Next to my work, I have enough resources (such as time and energy) left for my non-work life’. These items were scored on a 5-point Likert-scale ranging from 1 = *completely disagree* to 5 = *completely agree*. The two items were averaged to obtain an overall score. The internal consistency was good (*α = .84*).

#### Family-life stage (FLS)

Following Hill et al. [5], family-life stages were operationalized using participants’ age, number of children living at home and age of the youngest child living at home. Participants were categorized into five family-life stages.

- *Family-Life Stage 1* – participants 25 to 34 years old, no children
- *Family-Life Stage 2* – participants 20 years or older, youngest child preschool age
- *Family-Life Stage 3* – participants 25 years or older, youngest child in primary school
- *Family-Life Stage 4* – participants 30 years or older, youngest child older than 12
- *Family-Life Stage 5* – participants 55 years or older, no more children living at home.

*Level of employment* was measured in five categories: a) 100 %, b) 80–99 %, c) 50–79 %, d) 30 % 49 % and e) less than 30 %. In Switzerland, working full time (100 %) means 40 to 42 h per week. Because only few men worked less than 80 %, the categories were collapsed into a new bivariate variable with the categories ‘full time’ (100 %) and ‘part time’ (less than 100 %) for all analyses of variance.

*Relationship status* was used as a control variable and operationalized as an ordinal measure with three categories, 1 = “no partner”, 2 = “partner, no shared household”, 3 = “partner, shared household”.

### Analyses

A series of ANOVAs and hierarchical regression analyses were performed in order to answer the study’s research questions. For the ANOVAs, homogeneity of variances was assumed when Levene’s test was non-significant or when the variance ratio between the biggest and the smallest group variance was smaller than 2 [41]. This was true for all performed analyses of variance and will not be reported separately in the results section. In addition we conducted separate regression analyses for men and women to further explore the influence of level of employment on our dependent variables. This was done because gender and level of employment is highly correlated and the dichotomization into part- and full-time work for the analyses of variance does not accommodate for that. In these analyses, we controlled for relationship status.

## Results

### Descriptive analysis

The sample consisted of 2204 men and 1460 women (total *N* = 3664). The number of men and women per FLS can be seen in Table 2. The median age category was ‘36 to 40’ years. The age category ‘31 to 35’ held the highest absolute number of participants. 76.2 % of participants were living in a shared household with their partner. 8.2 % of participants had a partner but didn’t live in the same household with him/her, 14.1 % said, they had no partner and 1.5 % didn’t reply. 43.6 % of participants had no children living in the same household with them, and 56.4 % had one or more children living in the same household. 0.1 % did not reply but fit the criteria of family-life stage 1 otherwise.

Means, standard deviations, correlations and internal consistencies, where appropriate, of the central study variables are given in Table 1.

**Table 1:** Correlations, means, standard deviations and internal consistencies of study variables

Variables	2	3	4	5	6	M	SD	α
1 work demands	.20**	−.42**	−.14**	.19**	.07**	3.52	.82	
2 private demands		−.11**	−.01	−.12**	.05*	2.98	.91	
3 work-life balance			.10**	−.25**	.00	3.24	.93	.84
4 gender				−.50**	−.17**			
5 level of employment					.00			
6 relationship status								

*p < .01, ** *p* < 0.001, *n* = 3606–3664, gender: 0 = male, 1 = female

Table 2 gives the absolute and relative numbers of men’s and women’s level of employment. Most men worked full time across all FLS, with the highest rates in FLS 2, 3 and 4. While most women worked full time in FLS 1 (before they have children), most worked part time in all other family-life stages.

**Table 2:** Level of employment by gender and family-life stage

		FLS 1		FLS 2		FLS 3		FLS 4		FLS 5	
Level of employment		*n*	%	*n*	%	*n*	%	*n*	%	*n*	%
Men	100 %	559	86.3	406	90.6	547	93.3	345	95.0	139	87.4
	80 + %	56	8.6	36	8.0	31	5.3	16	4.4	11	6.9
	50 + %	19	2.9	5	1.1	3	0.5	1	0.3	5	3.1
	30 + %	10	1.5	1	0.2	2	0.3	0	0.0	2	1.3
	<30 %	4	0.6	0	0.0	3	0.5	1	0.3	2	1.3
Total		648	100	448	100	586	100	363	100	159	100
Women	100 %	478	72.5	33	14.3	41	15.4	34	18.9	37	30.3
	80 + %	139	21.1	29	12.6	40	15.0	47	26.1	31	25.4
	50 + %	26	3.9	97	42.2	96	36.0	71	39.4	45	36.9
	30 + %	12	1.8	50	21.7	63	23.6	27	15.0	8	6.6
	<30 %	4	0.6	21	9.1	27	10.1	1	0.6	1	0.8
Total		659	100	230	100	268	100	181	100	122	100
*n* per life-stage		1307		678		854		544		281	

*FLS* family-life stage

### Tests of hypotheses

#### Hypothesis 1: work demands

We first conducted a 5 × 2 × 2 ANOVA (FLS × gender × level of employment) with work demands as the dependent variable. The analysis yielded significant main effects for family-life stage (*F*_(4, 3642)_ = 3.70, *p* = .001, *η*_*p*_^*2*^ = .004) and level of employment (*F*_(1, 3642)_ = 18.37, *p* < .001, *η*_*p*_^*2*^ = .005). Further, the two-way interaction family-life stage x gender (*F*_(4, 3642)_ = 3.51, *p* = .007, *η*_*p*_^*2*^ = .004) was significant and the three-way interaction was significant, too (*F*_(4, 3642)_ = 2.73, *p* = .028, *η*_*p*_^*2*^ = .003).

Hypothesis 1a predicts that work demands will be highest in FLSs 2 and 3. In order to test this hypothesis, the main effect of FLS was explored. Bonferroni corrected post-hoc tests and Hochberg’s GT 2 tests showed that FLSs 2, 3 and 4 reported the highest work demands (*M =* 3.52, *SD =* .81; *M =* 3.58, *SD =* .82 and *M =* 3.60, *SD =* .82 respectively). These means all significantly differed from the work demands reported in FLS 5 (*M =* 3.35, *SD =* .91). FLS 1 (*M =* 3.49, *SD =* .79) differed significantly from FLS 3. These results mostly support hypothesis 1a, which states that work demands are highest in FLSs 2 and 3. The effect of FLS and the individual mean differences are rather small though.

**Fig. 1:**
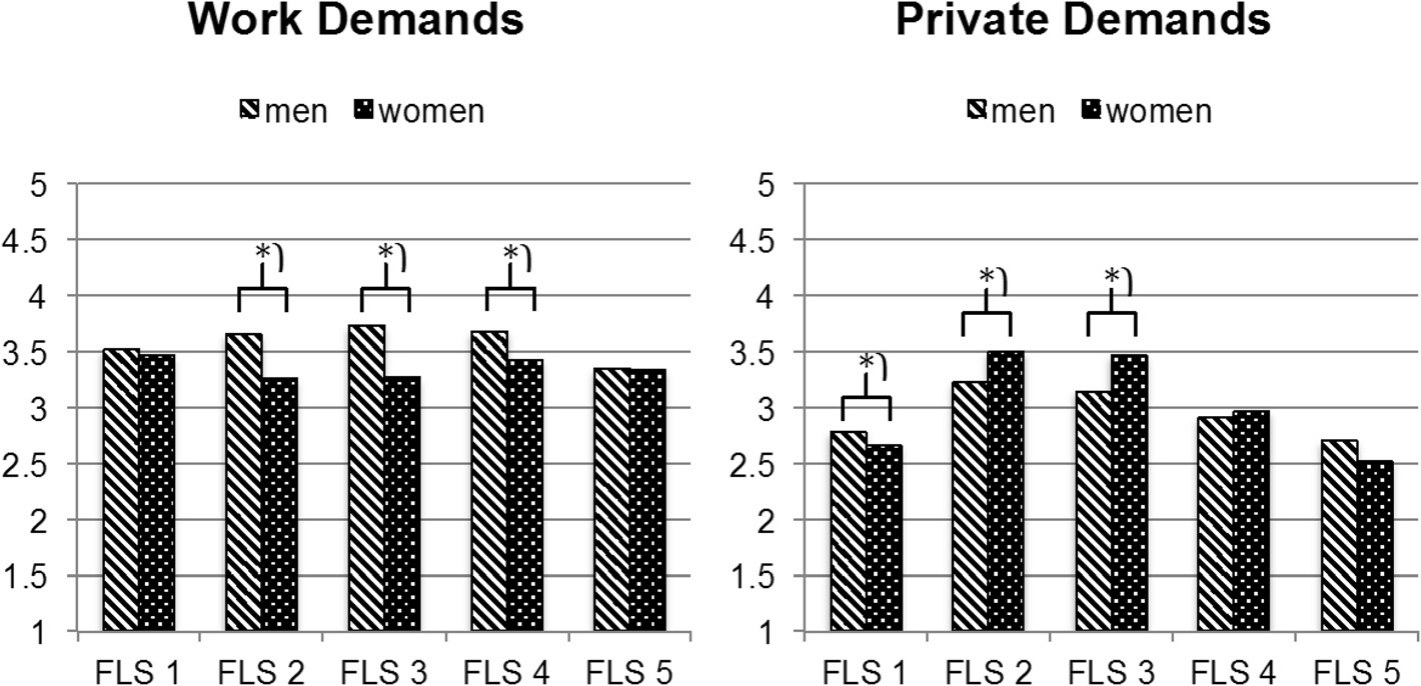
Interaction effects of family-life stage and gender on work and private demands

We then explored the FLS x gender two-way interaction effect, which was in line with hypothesis 1b. A one-factorial ANOVA with gender as the independent variable and work demands as the dependent variable was conducted for every FLS. To account for alpha accumulation, only *p*-values smaller than .01 were considered significant. Significant main effects for gender were found in FLS 2 (*F*_(1, 676)_ = 37.74, *p* < .001, *η*_*p*_^*2*^ = .053), FLS 3 (*F*_(1, 852)_ = 60.47, *p* < .001, *η*_*p*_^*2*^ = .066) and FLS 4 (*F*_(1, 542)_ = 11.99, *p* = .001, *η*_*p*_^*2*^ = .022). Men reported significantly higher work demands than women in FLS 2 (men: *M =* 3.65, *SD =* .76; women: *M =* 3.26, *SD =* .84), FLS 3 (men: *M =* 3.72, *SD =* .75; women: *M =* 3.27, *SD =* .88) and FLS 4 (men: *M =* 3.68, *SD =* .75; women: *M =* 3.43, *SD =* .91). The significant three-way interaction was not in line with our hypotheses. Conducting a gender x level of employment ANOVA for every FLS yielded a significant two-way interaction for FLS 1 only (*F*_(1, 1303)_ = 9.07, *p* = .003, *η*_*p*_^*2*^ = .007). Because hypothesis 2b predicts gender differences for FLSs 2 and 3 and does not make specific predictions for FLS 1, we did not further investigate this three-way interaction. To sum up, we found gender differences in FLS 2 and 3, which is in line with hypothesis 2b. Furthermore, there was a significant gender effect in FLS 4. Figure 1 illustrates the FLS x gender interaction effects on work demands.

To account for the fact that our part-time vs. full-time dichotomization means something very different for men and for women in the primary child-rearing family-life stages (women in part-time work work much fewer hours on average than men in part-time work), we further conducted hierarchical regression analyses for work demands with level of employment as a continuous predictor variable and relationship status as a control variable. We calculated separate regression equations for men and for women in FLS 2, 3 and 4. We found small effects (ΔR^2^ = .02 to .06) of level of employment on subjective work demands. Level of employment significantly predicted men’s subjective work demands in FLS 2 and women’s in FLS 2 and 3. Because of space limits, please refer to Table 3 for statistical details.

**Table 3:** Results of regression analyses

			Family-life stage 2				Family-life stage 3				Family-life stage 4			
			b	SEb	β	ΔR^2^	b	SEb	β	ΔR^2^	b	SEb	β	ΔR^2^
Work demands	m	Partner	.15	.15	.05	.00	.15	.10	.06	.00	.38	.11	.17*	.03*
		Partner	.10	.15	.03	.02*	.15	.10	.06	.00	.38	.11	.17*	.01
		Level employ.	.26	.10	.13*		.08	.07	.04		.23	.13	.09	
	f	Partner	−.15	.09	−.11	.01	.00	.07	.00	.00	.02	.09	.02	.00
		Partner	−.09	.09	−.07	.03*	.06	.07	.05	.06*	.05	.09	.04	.01
		Level employ.	.14	.05	.19*		.17	.05	.24*		.09	.07	.10	
Private demands	m	Partner	−.22	.17	−.06	.00	−.06	.13	−.02	.00	−.16	.13	−.07	.00
		Partner	−.19	.17	−.05	.00	−.06	.13	−.02	.00	−.16	.13	−.07	.00
		Level employ.	−.14	.11	−.06		−.09	.09	−.04		.06	.14	.02	
	f	Partner	−.04	.04	−.04	.00	−.05	.08	−.04	.00	−.03	.09	−.02	.00
		Partner	−.04	.04	−.04	.00	−.06	.09	−.05	.00	.02	.09	.01	.02*
		Level employ.	−.06	.05	−.05		−.01	.05	−.02		.14	.07	.15*	
Work-life balance	m	Partner	.17	.19	.04	.00	−.02	.12	−.01	.00	.03	.14	.01	.00
		Partner	.24	.19	.06	.02*	−.02	.12	−.01	.01	.03	.14	.01	.02*
		Level employ.	−.32	.12	−.13*		−.15	.09	−.07		−.35	.15	−.12*	
	f	Partner	.18	.10	.12	.01	.16	.08	.13	.02	.29	.09	.23*	.05*
		Partner	.00	.09	.00	.25*	.05	.07	.04	.19*	.16	.09	.12	.19*
		Level employ.	−.43	.05	−.51*		−.36	.05	−.45*		−.45	.07	−.45*	

*m* men, *f* women, *p < .05, partner = relationship status, Level employ. = Level of employment

#### Hypothesis 2: private demands

A 5 × 2 × 2 ANOVA (FLS × gender × level of employment) was performed with private demands as the dependent variable. The analysis yielded a significant main effect for family-life stage (*F*_(4, 3642)_ = 45.35, *p* < .001, *η*_*p*_^*2*^ = .047) and a significant two-way interaction for family-life stage x gender (*F*_(4, 3642)_ = .82, *p* = .001, *η*_*p*_^*2*^ = .005).

Hypothesis 2a predicts private demands to be highest in FLSs 2 and 3. In order to test this hypothesis, the main effect of FLS was explored. Bonferroni corrected post-hoc tests and Hochberg’s GT 2 tests showed that subjects in FLSs 2 and 3 reported the highest private demands (*M =* 3.32, *SD* = .84 and *M =* 3.25, *SD* = .91 respectively). The means of FLSs 2 and 3 differed significantly from the mean reported by subjects in FLS 4 (*M =* 2.93, *SD* = .86). Participants in FLSs 1 and 5 reported significantly lower private demands (*M =* 2.72, *SD* = .86 and *M =* 2.62, *SD* = .87 respectively). All *p*-values for significant mean comparisons were *p* < .001. This is in line with hypothesis 1a.

In order to explore the nature of the significant two-way interaction, a one-factorial ANOVA with gender as the independent variable and private demands as the dependent variable was conducted for every family-life stage. To account for alpha accumulation, only *p*-values smaller than .01 were considered significant. Significant main effects for gender were found in FLS 1 (*F*_(1, 1305)_ = 5.94, *p* = .015, *η*_*p*_^*2*^ = .005), FLS 2 (*F*_(1, 676)_ = 16.19, *p* < .001, *η*_*p*_^*2*^ = .023) and FLS 3 (*F*_(1, 852)_ = 24.02, *p* < .001, *η*_*p*_^*2*^ = .027). In FLS 1, private demands were higher for men (*M =* 2.78, *SD* = .83) than for women (*M =* 2.66, *SD* = .89). In line with hypothesis 2b, we found gender effects in FLS 2 and 3. Women reported significantly higher private demands than men (FLS 2: men *M =* 3.23, *SD* = .85; women *M =* 3.50, *SD* = .79; FLS 3: men *M =* 3.15, *SD* = .92; women *M =* 3.47, *SD* = .84). Figure 1 illustrates this interaction effect of FLS x gender.

Again we conducted hierarchical regression analyses for private demands with level of employment as a continuous predictor variable and relationship status as a control variable. We calculated separate regression equations for men and women in FLSs 2, 3 and 4. We only found tiny effects (ΔR^2^ = .02) of level of employment on subjective private demands for women in FLS 4. For statistical details, please refer you to Table 3.

#### Hypothesis 3: work-life balance

A 5 × 2 × 2 ANOVA (FLS x gender x level of employment) with work-life balance as the dependent variable was performed. The analysis yielded significant main effects for family-life stage (*F*_(4, 3642)_ = 8.06, *p* < .001, *η*_*p*_^*2*^ = .009) and level of employment (*F*_(1, 3642)_ = 78.22, *p* < .001, *η*_*p*_^*2*^ = .021) and two significant two-way interactions (gender x level of employment: *F*_(1, 3642)_ = 8.20, *p* = .004, *η*_*p*_^*2*^ = .002; family-life stage x level of employment: *F*_(4, 3642)_ = 5.12, *p* < .001, *η*_*p*_^*2*^ = .006). Furthermore, as predicted in Hypothesis 4b, the three-way interaction was significant (*F*_(4, 3642)_ = 2.90, *p* = .021, *η*_*p*_^*2*^ = .003).

Hypothesis 3a predicts work-life balance to be lowest in FLSs 2 and 3. In order to test this hypothesis, the main effect of FLS was explored. Bonferroni corrected post-hoc tests and Hochberg’s GT 2 tests showed that FLS 5 (*M =* 3.64, *SD =* .86) reported significantly higher work-life balance than all other FLSs (FLS 1: *M =* 3.19, *SD =* .92; FLS 2: *M =* 3.24, *SD =* .95; FLS 3: *M =* 3.19, *SD =* .92; FLS 4: *M =* 3.27, *SD =* .92). This result does not support our hypothesis. In line with hypothesis 3b, the main effect of FLS was qualified by a significant three-way interaction. A 2 × 2 ANOVA (gender x level of employment) for every FLS with work-life balance as the dependent variable yielded significant two way interactions for FLS 2 (*F*_(1, 674)_ = 7.56, *p* = .006, *η*_*p*_^*2*^ = .011) and FLS 3 (*F*_(1, 849)_ = 6.36, *p* = .012, *η*_*p*_^*2*^ = .007). Simple effects analyses for FLS 2 showed that men (*M =* 3.08, *SD =* .92) and women (*M =* 2.71, *SD =* .75) who work full time did not differ by their reported work-life balance, but men and women who work part time did differ significantly (*F*_(1, 673)_ = 19.05, *p* < .001). Women who work part time reported higher work-life balance than men who work part time (men: *M =* 3.37, *SD =* .88; women: *M =* 3.62, *SD =* .93). Furthermore, men who work part or full time did not differ in their reported work-life balance, while for women the predicted difference between part- and full-time workers was found (*F*_(1, 673)_ = 35.95, *p* < .001).

**Fig. 2:**
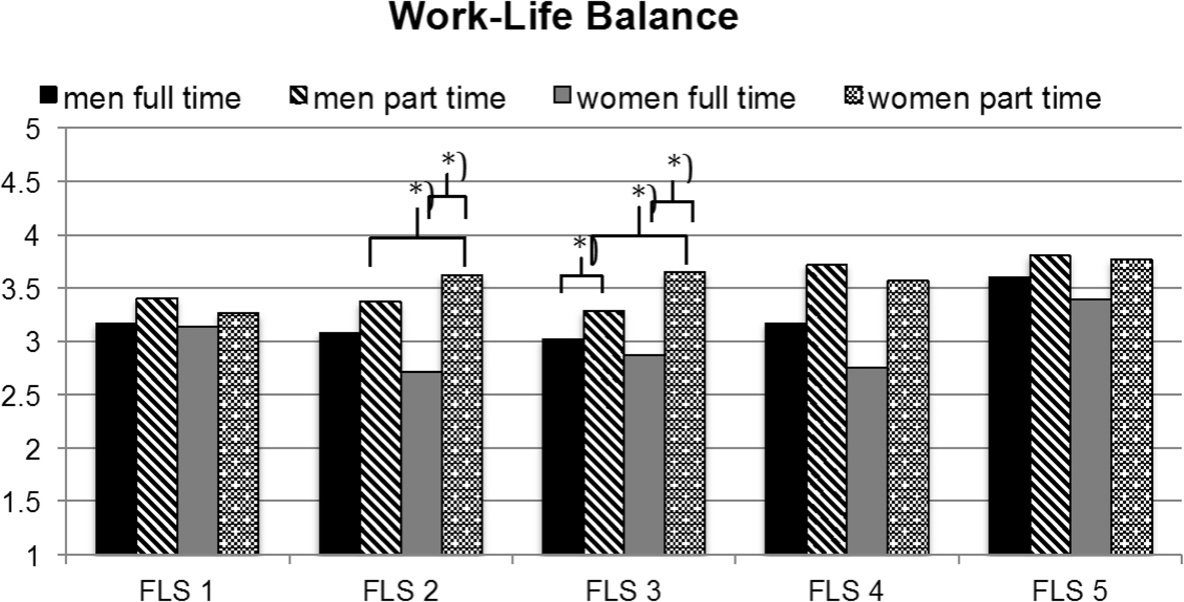
Three-way interaction effect of family-life stage, gender and level of employment on work-life balance

Simple effects analyses [41] for FLS 3 showed that men (*M =* 3.03, *SD =* .87) and women (*M =* 2.87, *SD =* .89) who work full-time did not differ in work-life balance, while men and women who work part time differed significantly (*F*_(1, 848)_ = 32.88, *p* < .001). Specifically, women who work part time reported higher work-life balance than men who work part time (men: *M =* 3.28, *SD =* .80; women: *M =* 3.65, *SD =* .91). Furthermore, there were significant differences in work-life balance between part- and full-time work for men (*F*_(1, 848)_ = 8.87, *p* = .003) and women (*F*_(1, 848)_ = 43.71, *p* < .001). The latter was predicted in hypothesis 4b. Figure 2 illustrates the gender by level of employment interaction effects on work-life balance in family-life stages 2 and 3.

We conducted hierarchical regression analyses for work-life balance with level of employment as a continuous predictor variable and relationship status as a control variable and calculated separate regression equations for men and women in FLSs 2, 3 and 4. We found that level of employment was a substantial predictor (ΔR^2^ = .19 to .25) of work-life balance for women in FLSs 2, 3 and 4. For men, we only found very small effects (ΔR^2^ = .02) in FLS 2 and 4. For statistical details, please refer to Table 3.

## Discussion

Our aim was to investigate men’s and women’s subjective work and private demands across family-life stages, as well as the experience of work-life balance in a sample of Swiss employees, given the actual gendered division of paid und unpaid work in families in Switzerland. For this purpose we looked at the subjective experience of demands in the work and private domain and the experience of work-life balance as a function of family-life stage, gender and level of employment.

With regard to subjective work demands, we expected the demands to be highest during family-life stages 2 and 3, because the career building years tend to coincide with the primary child rearing family-life stages. We further expected men to experience higher subjective work demands due to their role as primary breadwinners and their higher levels of employment. Our findings support this hypothesis. Participants in family-life stages 2, 3 and even 4 reported the highest work demands. This is in line with a study by Erickson et al. [27] that finds increases in work role demands across the primary child-rearing family-life stages for a big international sample of professionals. In addition, we found that men experience significantly higher work demands than women in those family-life stages. Additional analyses for men and women showed that the effect of level of employment on subjective work demands was rather small. We found an effect for men in FLS 2 and for women in FLS 2 and 3. Our interpretation of these findings is that work is experienced as rather demanding during the primary child-rearing family-life stages, that men are burdened more than women with work demands and that the extent to which work is experienced as demanding is only loosely tied to level of employment when looking at men and women separately. This could reflect the fact that men often do not have the possibility to work reduced hours and if they do, they might still be expected to get the same amount of work done as if they would work full time. In the case of women, these findings might reflect their second earner status within most couples. They might feel less burdened by paid work because they feel less responsible for the family’s financial security or they might encounter different expectations from their employers than men. It might also reflect a strategic selection of women, a strategy to choose less demanding jobs in order to take on more private responsibilities. There is evidence for this kind of strategic selection [42–44].

In the case of private demands our prediction was supported, too. Subjective private demands were highest in family-life stages 2 and 3 and women in those stages reported significantly higher demands than men. The level of employment had no influence on how burdened parents in family-life stages 2 and 3 felt by their private commitments. We interpret this finding in the sense that private demands cannot be regulated according to level of employment. Studies have shown that women not only spend more hours in childcare but also feel more responsible for care tasks while fathers’ time spent with children involves more play [45, 46]. Consequently, women in the primary child-rearing family-life stages might feel more burdened with the demands of the private domain than men. Together, our results for subjective global work and private demands mirror the actual gendered division of hours in paid and unpaid work in Swiss families [9].

We expected work-life balance to be lowest in family-life stages with small children, due to highest total demands in the work and the private domain. This was not confirmed, however. Work-life balance was best in family-life stage 5, when children are grown and have left home. All other family-life stages did not differ in terms of reconcilability of work and non-work life. We can conclude that the results for work-life balance don’t mirror the curvilinear relationship between family-life stages and work-life conflict that has been reported by previous studies [27, 29]. However, in line with hypothesis 3b, we found evidence for our assumption that women’s part time work affords them a better work-life balance in family-life stages 2 and 3. With regard to men, the evidence was less conclusive. The analysis of variance suggested a small effect of level of employment in family-life stage 4, while the results from the regression analyses found evidence for a positive effect of part-time work on work-life balance in family-life stage 3. Taken together we can conclude, that despite very high overall demands in family-life stages with small children at home, women can maintain a better work-life balance by working low to moderate levels of part time. Having said that, women who work full time and men, for whom full-time work is the norm and part-time work an exception, find it harder to reconcile work and non-work life in family-life stages with small children; they report lower levels of work-life balance.

### Strengths and limitations and future research

Even though our sample might not be representative for the entire working population, it covers a wide variety of jobs in divers sectors across hierarchy levels. Further research including different samples is needed in order to generalize our findings. Specifically, blue collar workers are underrepresented in our sample and might show different patterns of work demands over the life course. Our study needs to be replicated for this population of workers before findings can be generalized.

We used cross-sectional data in this study and therefore the effects of family-life stages might be confounded with cohort effects. Longitudinal studies are necessary to disentangle life-course effects from cohort effects.

Despite these limitations, we believe that our study adds to the existing research on the gendered division of labor in Switzerland by highlighting that men’s and women’s subjective experience of work-life balance depends on the interplay of life stage and level of employment. Future research should investigate whether there are life stage specific predictors of work-life balance. Furthermore, there might be life stage-, gender- and level of employment-specific consequences of work-life balance, such as consequences for life satisfaction, health and well-being.

Since part-time work is not so common among men with small children at home, we had relatively few fathers in our dataset who worked reduced hours. Future research should focus on fathers with reduced hours and investigate whether their subjective work-life balance benefits as much from part-time work as women’s.

## Conclusions

Our data illustrates fathers’ and mothers’ subjective experience of the gendered division of labor. The psychological experience of work and private demands parallels the actual division by hours [9]. It illustrates the inequality of men and women in the work and the private realm – and in its consequence a health inequality – on a more experiential level. According to Swiss statistics [24], in 2012 13.7 % of mothers who worked part time said they would like to work more. 37.8 % of mothers who worked full time said they would like to reduce their hours. Among fathers who worked full time, 18.4 % said they would prefer part-time work and only 1.5 % of part-time working fathers wanted to increase their hours. These findings together with our findings for fathers’ and mothers’ unequal experience of work-life balance as a result of their gendered division of paid and unpaid work speaks to a “fundamental mismatch” (Moen, 2011; pp. 87) between today’s workforce and todays workplace. As work-life balance is an indicator of satisfaction with the extent to which people feel they live in accordance with their current life values, our results make it clear that more state support and workplace flexibility in terms of part-time work for men is sorely needed in order to enable today’s parents to create a more equitable experience in the work and the private domain. Because research has repeatedly shown that that work-life balance is related to physical and psychological health indicators, it can be argued that the promotion of work-life balance is of importance in light of public health considerations.
